# Univariate and multivariate signal processing spectrophotometric determination of an antihypertensive combination in line with the United Nations sustainable development goals

**DOI:** 10.1038/s41598-025-22700-0

**Published:** 2025-10-31

**Authors:** Mona A. Kamel, Hoda M. Marzouk, Adel M. Michael, Samah S. Abbas, Christine K. Nessim

**Affiliations:** 1 Postgraduate Program, in Pharmaceutical Analytical Chemistry, Faculty of Pharmacy, Cairo University, Kasr El-Aini Street, Cairo, 11562 Egypt; 2 Chemistry Department, Faculty of Pharmacy, Ahram Canadian University, 6th of October City, Cairo, 12566 Egypt; 3 Pharmaceutical Analytical Chemistry Department, Faculty of Pharmacy, Cairo University, Kasr El-Aini Street, Cairo, 11562 Egypt; 4 Charles Sturt University, Wagga Wagga, Australia

**Keywords:** Amlodipine, Chlorthalidone, GAPLS, iPLS, Sustainability (NQS) index, Telmisartan, Chemistry, Mathematics and computing

## Abstract

Effective management of hypertension typically involves multiple medications. This underscores the pharmaceutical industry’s demand for simple, cost-effective, and environmentally sustainable analytical methods capable of handling complex, multicomponent formulations. This study’s primary goal was to compare and validate univariate and multivariate spectrophotometric techniques for analyzing fixed-dose antihypertensive formulations of Telmisartan (TEL), Chlorthalidone (CHT), and Amlodipine (AML). Successive Ratio Subtraction paired with Constant Multiplication (SRS-CM) and Successive Derivative Subtraction paired with Constant Multiplication (SDS-CM) were the developed univariate methods. The cited drugs were successfully quantified at their respective maxima: 295.7 nm for TEL, 275.0 nm for CHT, and 359.5 nm for AML. On the other hand, the SDS-CM method enabled their determination using first-derivative spectra, with TEL identified at P_282.5–313_ nm, CHT at 287.0 nm, and AML at P_231-246_ nm. Also, Interval-Partial Least Squares (iPLS) and Genetic Algorithm-Partial Least Squares (GA-PLS) were applied as multivariate techniques. In contrast to full-spectrum modeling alone, the results showed that adding variable selection techniques greatly improved the model’s performance. Following ICH guidelines, the proposed techniques were used to quantify the cited medications in tablets. The validity of the results was confirmed by statistical comparison with the reported method. The study was further expanded to assess the content uniformity of the dosage units in compliance with USP. Three environmental complementary assessment tools were employed: the Analytical Greenness Metric (AGREE), the Blue Applicability Grade Index (BAGI) and White Analytical Chemistry (RGB12). This study also aligns with several UN Sustainable Development Goals (UN-SDGs), emphasizing commitment to green pharmaceutical research. Sustainability was verified using the NQS index, confirming the method’s compliance with responsible analytical practices.

**Supplementary Information:**

The online version contains supplementary material available at 10.1038/s41598-025-22700-0.

## Introduction

One of the main risk factors for cardiovascular disease is hypertension. According to estimates from the World Health Organization (WHO), high blood pressure causes at least nine million deaths worldwide annually^1^. A combination of therapeutic agents acting through synergistic mechanisms is generally preferred to maximize therapeutic efficacy while minimizing adverse effects, even though single-tablet regimens (STRs) for the treatment of hypertension are linked to better quality of life, increased medical adherence, and higher patient satisfaction when compared to multi-tablet regimens (MTRs). Administering these agents as a fixed-dose combination in a single tablet or capsule is particularly advantageous, as it enhances patient adherence and compliance, especially among geriatric populations^1^. A promising and successful strategy for the early management of hypertension that strikes a balance between safety and effectiveness is low-dose combination therapy^2^.

The most often suggested combinations for treating hypertension are an angiotensin-converting enzyme inhibitor or an angiotensin receptor blocker combined with a calcium channel blocker, following US^3^ and European^4^, recommendations. A thiazide-like diuretic can be added as part of a triple combination therapy to improve therapeutic outcomes for patients whose blood pressure is still uncontrolled. The medication combination of TEL, CHT and AML is used to treat hypertension and is prepared as a single tablet regimen^5^. Both the British Pharmacopeia^6^ and the United States Pharmacopeia^7^ list these medications.

**Fig. 1:**
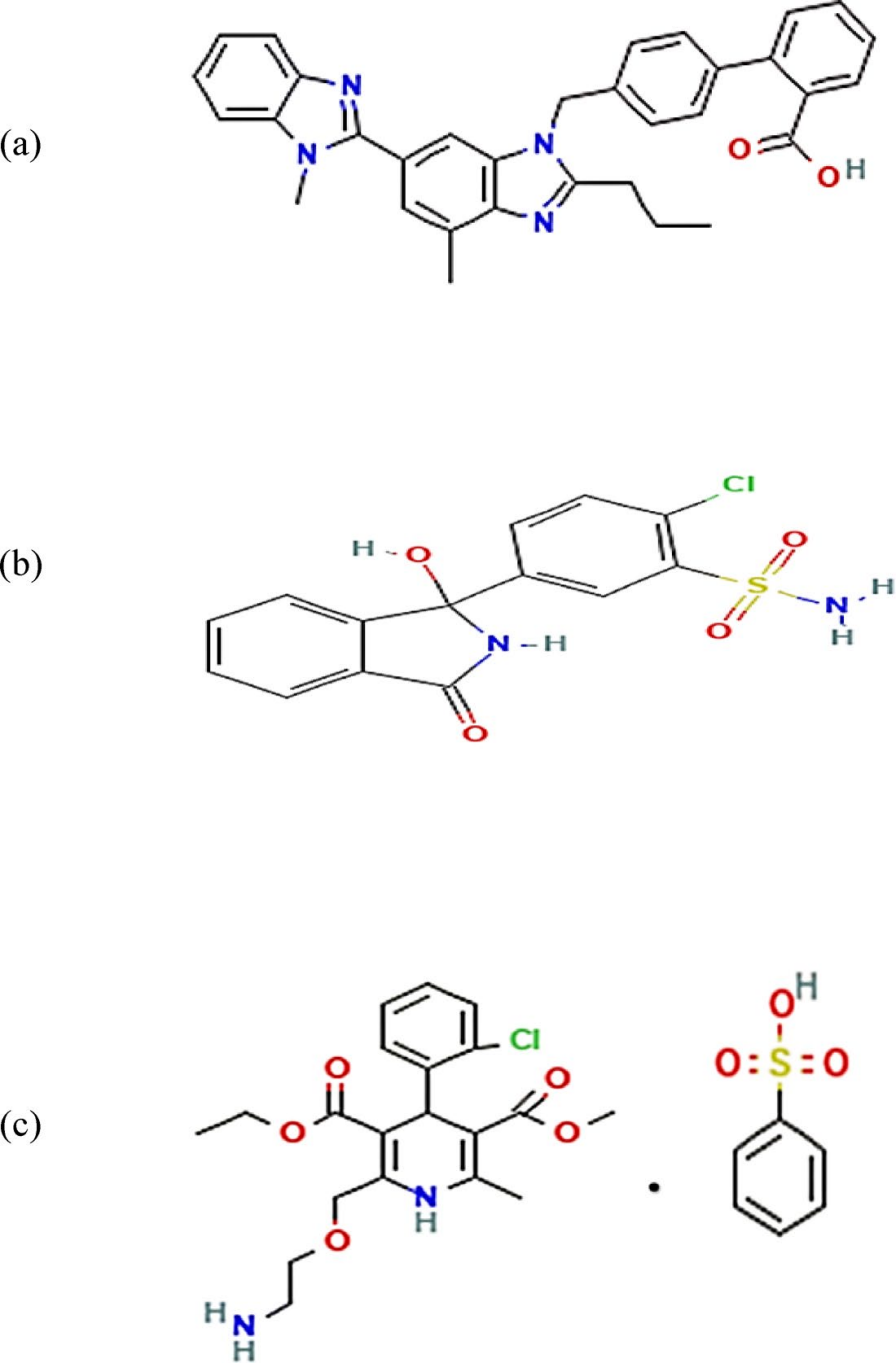
Chemical structures of (**a**) Telmisartan (TEL), (**b**) Chlorthalidone (CHT) and (**c**) Amlodipine besylate (AML).

TEL (Fig. 1a) is 4′-[[4-Methyl-6-(1-methyl-1H-benzimidazol-2-yl)-2-propyl-1H-benzimidazol-1- yl] methyl] biphenyl-2-carboxylic acid^6^. TEL selectively and reversibly binds to the angiotensin II type 1 (AT1) receptor to act as an angiotensin II receptor blocker (ARB). It suppresses the angiotensin II-stimulated production of aldosterone and encourages vasodilation by blocking the AT1 receptor^8,9^. CHT (Fig. 1b), chemically known as 2-Chloro-5-[(1RS)-1-hydroxy-3-oxo-2,3-dihydro-1H-isoindol-1-yl] benzene sulfonamide^6^, is a long-acting thiazide-like diuretic. It is used to treat high blood pressure and edema associated with heart, kidney, or liver failure^10^. AML (Fig. 1c), a 1,4-dihydropyridine-3,5-dicarboxylate derivative^6^, functions as a calcium channel blocker by preventing the influx of calcium ions into vascular and cardiac muscles. Recognized by the World Health Organization (WHO) as an essential antihypertensive medication, AML is considered a highly safe and effective treatment for hypertension and chronic stable angina^11^.

The successive spectrophotometric resolution method can be utilized to resolve spectral overlap when analyzing multi-component mixtures, without requiring any prior separation processes^12,13^. Two variable selection techniques were used to improve the predictive power ability of PLS model including interval-PLS and Genetic Algorithm-PLS. Interval-PLS enhances both the clarity and accuracy of the model by focusing on the most relevant intervals, thereby reducing noise and the risk of overfitting^14–16^. Genetic Algorithm (GA) is an optimization method based on principles of natural evolution and genetics. It works by evolving a set of potential solutions through processes such as selection, crossover, and mutation. GA is especially effective for selecting features, optimizing parameters, and fine-tuning models in areas like machine learning and chemometrics^17^.

Reviewing the literature, only few reported methods were published for determination of the studied mixture including RP-HPLC^18,19^, HPTLC^20^, and UV spectrophotometry^21^. Also, few spectrophotometric and HPLC methods were published for cited drugs along with other antihypertensive drugs^22–24^. The reported spectrophotometric method^21^ that analyzed the same studied drug mixture employed absorption correction method using methanol as solvent. The wavelengths selected for the analysis of TEL, CHT and AML were 311, 228, and 253 nm, respectively. This reported method^21^ lacked an evaluation of its environmental impact.

In light of escalating environmental and health concerns linked to conventional analytical methodologies, there is a pronounced imperative to adopt green analytical chemistry (GAC). This study employs UV–Vis spectrophotometry, a technique aligned with the sustainability goals of modern analytical chemistry. The method is cost-efficient, utilizing affordable instrumentation and reagents while generating minimal hazardous waste, thereby adhering to the core tenets of GAC and White Analytical Chemistry (WAC)^25^. In this study, the used solvent was ethanol which is one of the green solvents due to its renewable sourcing, biodegradability, and low toxicity compared to conventional organic solvents. Four implemented assessment tools were used to evaluate the environmental sustainability and practical applicability of the proposed analytical methods. These methods were Analytical Greenness Metric (AGREE)^26^, and Blue Applicability Grade Index (BAGI)^27^. Additionally, White Analytical Chemistry (RGB12)^28^, and Need, Quality, Sustainability (NQS) index which are newly introduced assessment approaches^29^.

In this work, comparative studies were developed, incorporating both univariate and multivariate spectrophotometric methods for the simultaneous determination of TEL, CHT and AML in their mixture. To the best of our knowledge, the previously reported methods for this ternary mixture are limited to univariate spectrophotometric approaches only. Therefore, the first aim of this research was to develop two successive spectrophotometric resolution techniques including successive ratio subtraction and successive derivative subtraction, and both coupled with constant multiplication. The second goal was to develop comparative studies using multivariate chemometric approaches including (PLS) combined with two variable selection techniques: (iPLS) and (GA-PLS). Those proposed methods were successfully validated and applied for the analysis of TEL, CHT, and AML in their laboratory-prepared mixtures, fixed combination dosage form (Telma-ACT® Tablets) and for the evaluation of content uniformity of individual units addressing a critical quality control requirement. The third goal was to make ecological assessments of the proposed methods including AGREE, BAGI and RGB12. The study also supports multiple United Nations Sustainable Development Goals (UN-SDGs), particularly goals 3, 4, 5, 7, 9, 11, 12, 13, 14, 15, and 17, highlighting a strong commitment to sustainable pharmaceutical research^30^.

## Experimental

### Materials and reagents

Pure Telmisartan and Amlodipine Besylate, with certified purities of 99.58% and 98.75%, respectively, were generously provided by Global NAPI Pharmaceuticals, Egypt. Chlorthalidone, with a certified purity of 99.12%, was kindly supplied by EIPICO Pharmaceuticals, Egypt. Commercially available Telma-ACT® Tablets with a label claimed of 40.0 mg of TEL, 12.5 mg of CHT and 5.0 mg of AML per tablet (BN: 30TTM008) were obtained from a local Indian pharmacy. Ethanol (HPLC grade) was supplied from Sigma-Aldrich (Germany).

### Apparatus and software

Spectrophotometric assay was done using a double beam UV/Vis spectrophotometer model V-760, Jasco, Japan, using spectra manager® software connected to a compatible computer. The absorption spectra of the measured solutions were carried out in a 1.0 cm quartz cell in the range of 200.0 to 400.0 nm at room temperature.

All chemometric assisted models were carried out using Matlab R2024a (24.1.0.2628055) and PLS toolbox software version 9.3.1.

### Standard solutions

Three separate stock solutions of TEL, CHT, and AML (500.0 µg/mL each) were prepared by accurately weighing 50.0 mg of each pure analytical standard and transferring it into a 100-mL volumetric flask and completed to the mark with ethanol. The solutions were stirred for 10 min to ensure complete dissolution of the standards. These solutions were then protected from light and stored at a controlled temperature of 2–8 °C.

Fresh working solutions were prepared by precisely measuring 20.0 mL of the stock solutions into individual 100-mL volumetric flasks. Each flask was then filled to the calibration mark with ethanol to achieve a final concentration of 100.0 μg/mL for each drug. All standard solutions were stored in sealed, light-protected containers.

### Procedures

#### Univariate spectrophotometric methods

##### Calibration curves construction

Three separate sets of 10-mL volumetric flasks were used to accurately transfer multiple aliquots from the stock standard solutions of TEL, CHT, and AML to establish their respective linearity ranges using ethanol as diluting solvent: (5.0–40.0 µg/mL) for TEL, (10.0–100.0 µg/mL) for CHT, and for (5.0–25.0 µg/mL) AML.

*Successive ratio subtraction method coupled with constant multiplication method.* The zero-order absorption spectra of TEL, CHT, and AML were scanned separately at 200.0–400.0 nm and stored in the computer. The calibration curves were constructed and related a linear relationship between the absorption of TEL, CHT and AML at 295.7 nm, 275.0 nm and 359.5 nm, respectively versus their corresponding concentrations. Then the regression equation of each calibration curve was computed.

Successive Ratio Subtraction method was first applied to isolate the spectrum of the less extended drug (CHT) using spectrum of standard AML′ (10.0 µg/mL) as divisor I and spectrum of standard TEL′ (10.0 µg/mL) as divisor II. Subsequently, the Complementary Constant Multiplication method was employed to retrieve the spectra of the more extended drugs (TEL and AML), where recorded constants were multiplied by the absorption spectra of 10.0 µg/mL AML and TEL to reconstruct their respective zero-order spectra. Each drug’s concentration was then determined from the regression equation correlating absorbance values at their λ_max_ against corresponding concentrations.

*Successive derivative subtraction coupled with constant multiplication method.* The first derivative spectra (D^1^) (Δ λ = 10, scaling factor = 10 and subtract algorithm) of TEL, CHT, and AML were scanned at 200.0–400.0 nm and stored in the computer. The calibration curves were constructed and related a linear relationship between the peak amplitudes (peak-zero baseline or peak-peak) of the first derivative spectra of TEL, CHT and AML at P_282.5–313.0_ nm, 287.0 nm, and P_231.0–246.0_ nm, respectively versus their corresponding concentrations**.**

By applying successive derivative subtraction method, CHT concentration was obtained by dividing the D1 spectra of mixtures by D1 spectrum of standard AML′ (10.0 µg/mL), subtracting AML/AML′, multiplying by the D1 spectrum of standard AML′, then repeating the process using D1 spectrum of standard TEL′ (5.0 µg/mL) as divisor II to yield the pure first-derivative spectra of CHT. The first derivative spectra of AML and TEL were obtained using the constant multiplication method by multiplying the recorded constants with the D1 spectra of standard AML′ (10.0 µg/mL) and TEL′ (5.0 µg/mL), and their concentrations were calculated from regression equations relating peak amplitudes to corresponding concentration.

##### Application to the laboratory prepared mixtures

*Successive ratio subtraction method coupled with constant multiplication method.* Ratio spectra of laboratory-prepared mixtures were obtained using 10.0 µg/mL AML as a divisor. The amplitude of the constant AML/AML′(I) was recorded in the plateau region (355.0–385.0 nm). These constants were then subtracted from the ratio spectra, and the resulting spectra were multiplied by the spectrum of 10.0 µg/mL AML.

Subsequently, the obtained spectra were divided by the spectrum of 10.0 µg/mL TEL as a divisor. The amplitude of the constant TEL/TEL′(II) was recorded in the plateau region (300.0–321.0 nm), and these constant values were subtracted from the corresponding ratio spectra. The resulting spectra were then multiplied by the spectrum of 10.0 µg/mL TEL to obtain the pure zero-order spectra of CHT. The zero-order absorption spectra of AML and TEL were acquired using a complementary approach, the constant multiplication method. The recorded constant values (I) were multiplied by the absorption spectrum of the divisor, 10.0 µg/mL AML, to obtain the corresponding D^0^ spectrum of AML. Similarly, the recorded constant values (II) were multiplied by the absorption spectrum of the divisor, 10.0 µg/mL TEL, to obtain the corresponding D^0^ spectrum of TEL. The concentration of each compound was calculated using the regression equation correlating D^0^ at their respective λ_max_ values with their concentrations.

*Successive derivative subtraction coupled with constant multiplication method.* The D1 spectra of laboratory-prepared mixtures were recorded and divided by the D1 spectrum of AML (10.0 µg/mL), then the amplitudes of the constant AML/AML′(I) were recorded in the plateau region (375.0–390.0 nm) then subtracted. The obtained spectra were multiplied by the D1 spectrum of AML (10.0 µg/mL). The obtained spectra used for determination of TEL and CHT by dividing these spectra by the D1 spectrum of TEL (5.0 µg/mL) as divisor. Then the amplitudes of the constant TEL/TEL′(II) were recorded in the plateau region (305.0–325.0 nm) then subtracted. The obtained spectra were multiplied by the D1 spectrum of TEL (5.0 µg/mL) to obtain the pure first derivative spectra of CHT.

The first derivative spectra of AML and TEL were acquired using a complementary approach, the constant multiplication method. The recorded constant values (I) were multiplied by the D1 spectrum of the divisor, 10.0 µg/mL AML, to obtain the corresponding D1 spectrum of AML. Similarly, the recorded constant values (II) were multiplied by the first derivative spectrum of the divisor, 5.0 µg/mL TEL, to obtain the corresponding D1 spectrum of TEL.

The concentration of each compound was calculated using their regression equation correlating their concentration with their respective peak amplitudes (peak-zero baseline or peak-peak) of the first derivative spectra of TEL, CHT and AML at P_282.5–313.0 nm_, 287.0 nm, and P_231.0–246.0 nm_, respectively.

#### Multivariate spectrophotometric methods

##### Construction of the calibration model

A multilevel multifactorial design with three factors and five levels was employed to develop the calibration set. This set comprised seventeen laboratory-prepared mixtures with varying concentrations of TEL, CHT, and AML. These mixtures were meticulously prepared by combining and diluting accurately measured volumes from respective working solutions (100.0 μg/mL). The concentration ranges of the mixtures were 8.00–40.0 μg/mL for TEL, 10.0–90.0 μg/mL for CHT, and 5.0–25.0 μg/mL for AML. The absorption spectra of these mixtures were recorded over the range of 270.0–400.0 nm at 0.1 nm intervals. Subsequently, the data was exported and analyzed using Matlab equipped with the PLS toolbox to construct the calibration models.

##### Validation of calibration models

The developed models were subjected to both internal and external validation. Internal validation was conducted during the model-building process using cross-validation. For external validation, an independent set of eight mixtures containing the cited drugs was used to assess prediction accuracy and evaluate model performance.

#### Pharmaceutical formulation analysis

Twenty Telma-ACT® tablets (40.0 mg/12.5 mg/5.0 mg) were accurately weighed, and their mean weight was calculated. The tablets were then finely powdered, and an amount equivalent to 40.0 mg of TEL, 12.5 mg of CHT, and 5.0 mg of AML was weighed and transferred into a 100-mL volumetric flask. The flask volume was brought to the mark with ethanol, and the contents were sonicated for 30 min to ensure complete dissolution of the active ingredients. The solution was then filtered through Whatman filter paper. A 1-mL aliquot of the filtered solution was transferred to a 10-mL volumetric flask, which was then brought to volume with ethanol, yielding a testing solution containing 40.0 µg/mL of TEL, 12.5 µg/mL of CHT, and 5.0 µg/mL of AML. Applying the developed methods, the concentration of each drug was determined by applying the respective regression equations.

#### Content uniformity assessment of dosage form

The developed methods were additionally employed to evaluate the content uniformity of the marketed tablets in compliance with USP ^7^. The analysis followed the same procedures used for determining TEL, CHT, and AML in Telma-ACT® tablets, with the modification that a single intact tablet was analyzed per run. Absorption spectra were recorded, and the corresponding concentrations of the drugs were determined using the proposed methods. This process was repeated for ten individual tablets.

## Results and discussion

The reported univariate spectrophotometric method for the determination of the ternary mixture of TEL, CHT, and AML did not demonstrate direct applicability to the assay of the pharmaceutical dosage form nor the content uniformity. In contrast, the proposed univariate and multivariate methods were successfully applied for dosage form and content uniformity. Moreover, the proposed methods offered a wider linearity range with better reliability and proved to be environmentally-friendly. The use of ethanol in the proposed methods offers a safer and more environmentally friendly alternative to the hazardous organic solvents employed in previously reported spectrophotometric procedures. As a renewable and biodegradable solvent with low toxicity, ethanol enhances the green profile of the method while maintaining analytical performance.

### Univariate spectrophotometric methods

The zero-order and first derivative spectra of the cited drugs were severely overlapped and could not be determined directly in their mixtures, so two successive spectrophotometric resolution techniques were developed for solving the problem. These techniques were successive ratio subtraction and successive derivative subtraction.

#### Successive ratio subtraction method coupled with constant multiplication method

**Fig. 2:**
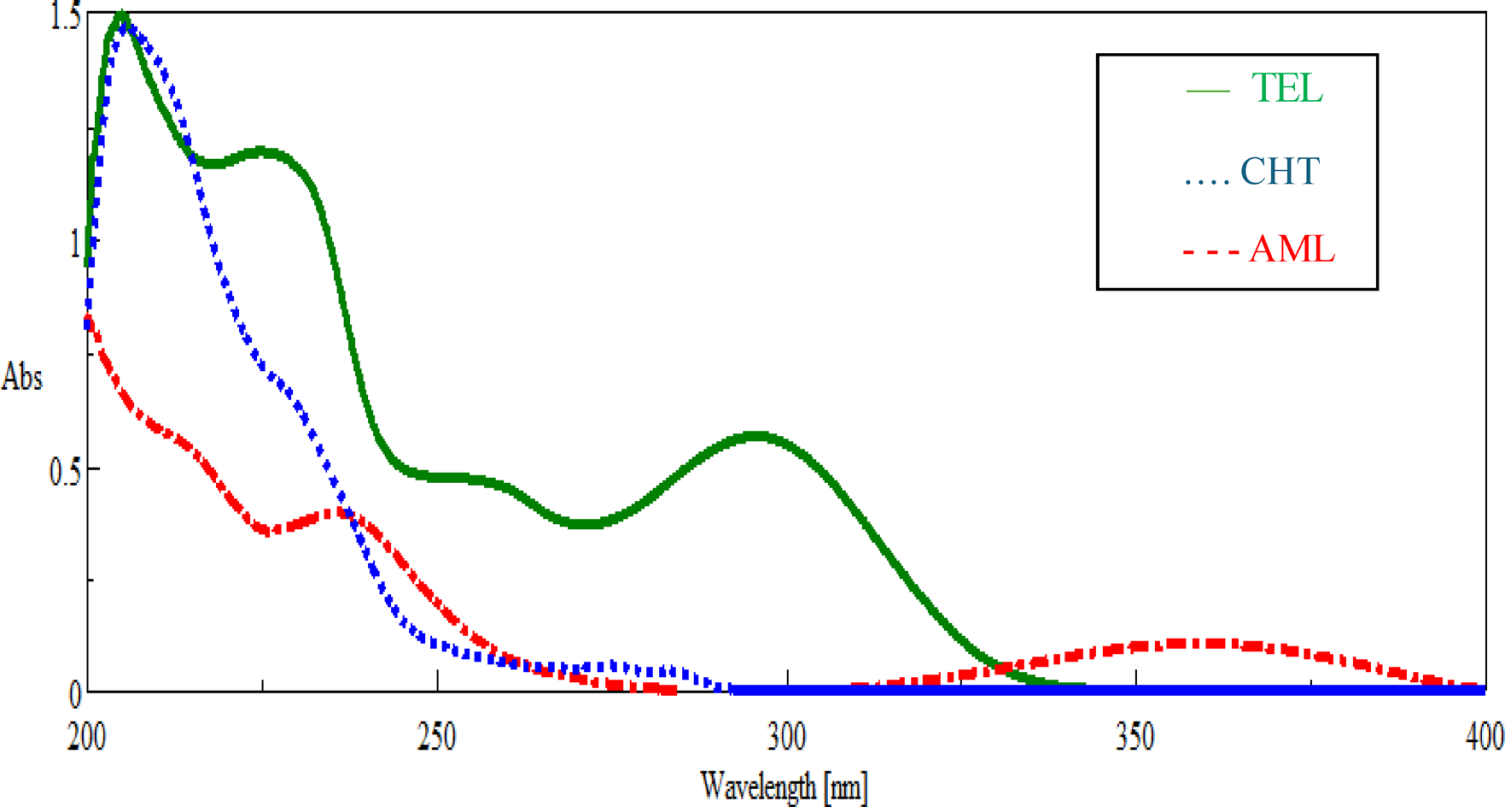
Zero-order spectra overlay of TEL , CHT , and AML  (10.0 µg/mL, each).

One of the advantages of applying this method was the ability of this method to cancel the whole spectrum of interfering substances, so the selected wavelength for calibration was the zero order λ_max_ of each drug. The overlaying zero order absorption spectrum of the three cited drugs showed that AML was more extended than TEL which was in turn more extended than CHT as shown in Fig. 2. By dividing the spectrum of laboratory prepared mixture by the divisor spectrum of AML (10.0 µg/mL), a constant-I was obtained from the straight line that was parallel to the wavelength axis at the extended region (AML/AML′) (355.0–385.0 nm). When multiplying the recorded constant-I by the divisor spectrum, the original zero order of corresponding AML was obtained. The AML absorbance was measured at its λ_max_ 359.5 nm.

1 $$\frac{{\left( {CHT + TEL + AML} \right)}}{{AML^{\prime } }} = \frac{CHT}{{AML^{\prime } }} + \frac{TEL}{{AML^{\prime } }} + \frac{AML}{{AML^{\prime } }} = \frac{CHT + TEL}{{AML^{\prime } }} + Constant\;I$$

2 $${\text{Constant}}\;{\text{I}} \times {\text{AML}}^{\prime } = {\text{AML}}$$

To eliminate AML spectrum, the constant-I was subtracted and then multiplied by the divisor spectrum, and the zero order spectrum of TEL and CHT was obtained.

3 $$\frac{CHT+TEL}{AML^{\prime}}+Constant\;I-constant\;I$$

4 $$\frac{CHT+TEL}{AML^{\prime}} x AML^{\prime}=\text{ CHT }+\text{ TEL}$$

The more extended zero spectrum of TEL was determined by dividing this spectrum by 10 µg/mL TEL′ as divisor. The obtained spectrum after division contained a plateau region TEL/TEL′ (constant-II) that was parallel to the wavelength axis at the extended region (300.0–321.0 nm). A multiplication of constant-II by the divisor (10 µg/mL TEL′), the original zero order of corresponding TEL was obtained. The TEL absorbance was measured at its λ_max_ 295.7 nm.

5 $$\frac{{\left( {CHT + TEL } \right)}}{{TEL^{\prime } }} = \frac{CHT}{{TEL^{\prime } }} + \frac{TEL}{{TEL^{\prime } }} = \frac{CHT}{{TEL^{\prime } }} + Constant\;II$$

6 $${\text{Constant}}\;{\text{II}} \times {\text{TEL}}^{\prime } = {\text{TEL}}$$

To eliminate TEL spectrum and obtain the original zero order spectrum of CHT, the constant-II was subtracted then the obtained spectrum was multiplied by the divisor spectrum TEL (10.0 µg/mL TEL′). The CHT absorbance was measured at its λ_max_ 275.0 nm. The steps that illustrating the method are presented in Fig. S1.

7 $$\frac{CHT}{TEL^{\prime}}+Constant\;II-constant\;II$$

8 $$\frac{CHT}{TEL^{\prime}} x TEL^{\prime} =\text{ CHT}$$

One of the most important parameters to be optimized for this method was selection of divisor’s concentrations. So different divisors’ concentrations of AML and TEL (5.0, 10.0, 20.0 µg/mL) were tried. It was found that 10.0 µg/mL gave the best results related to sensitivity and signal to noise ratio.

#### Successive derivative subtraction coupled with constant multiplication method

This technique can be used for resolving multicomponent mixtures with severely overlapping zero order absorption spectra using their derivative spectra. Through applying this method, we could obtain the pure D1 spectrum of each component in the mixture free from interferences by other components. Consequently, the peak-to-peak amplitudes of the D1 spectra for each drug can be accurately measured. This approach results in higher amplitude values, leading to steeper slopes and greater sensitivity. In contrast, when the derivative technique is applied without prior resolution, the amplitudes are measured less effectively, either at zero crossing points or at points of zero contribution from interfering substances (peak-to-baseline measurements).

**Fig. 3:**
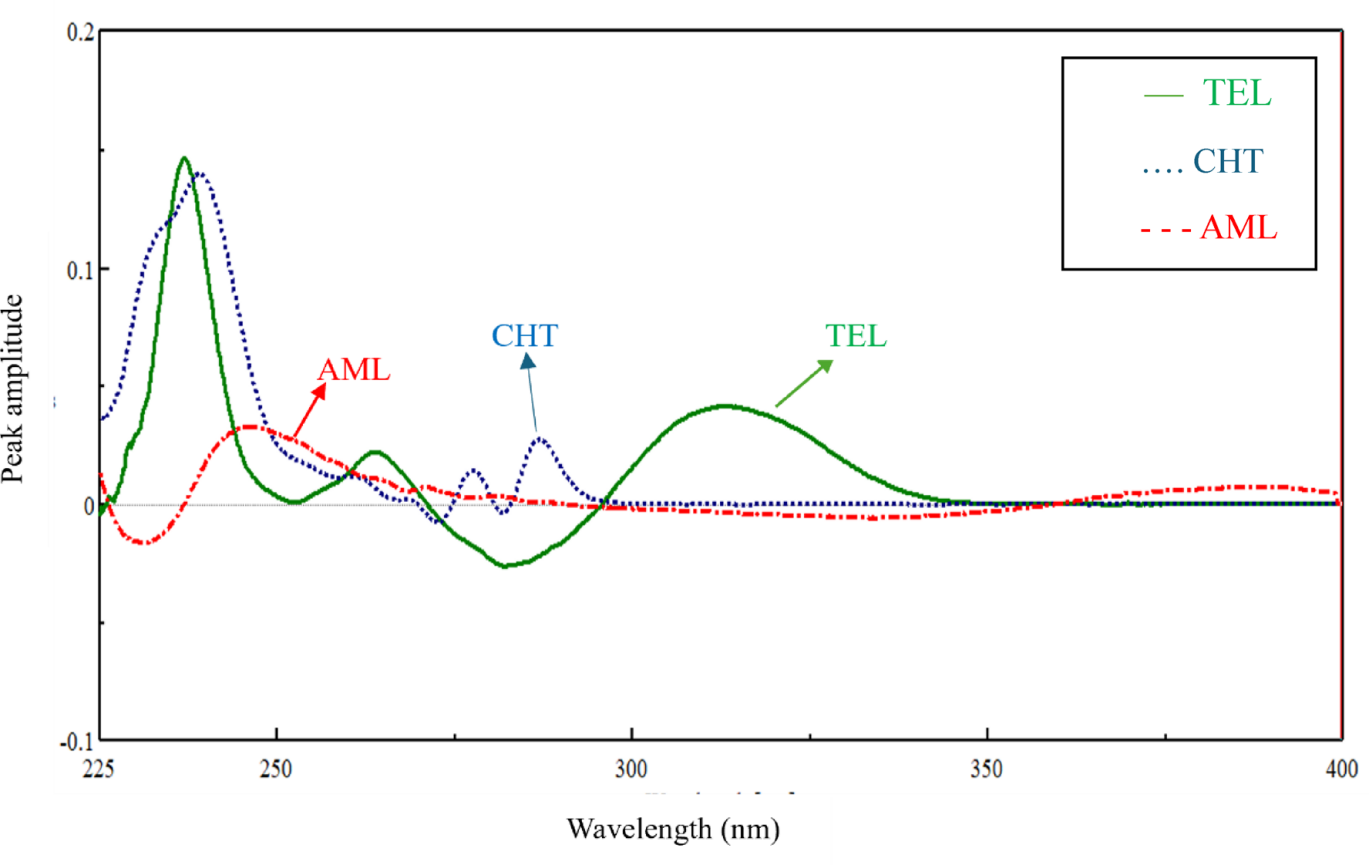
First derivative spectra of TEL (20.0 µg/mL) , CHT (40.0 µg/mL)  and AML (20.0 µg/mL) .

D1 spectra of three overlaying drugs showed that AML was more extended than TEL which was in turn more extended than CHT as shown in Fig. 3. D1 spectra of the laboratory prepared mixtures were divided by D1 spectra of AML (10.0 µg/mL), then value of constant I (AML/AML$^{\prime}$) was measured in the plateau region that was parallel to wavelength axis (375.0–390.0 nm). When multiplying the recorded constant-I by the divisor spectrum (D1 of AML$^{\prime})$, the original D1 of corresponding AML was obtained. The AML amplitude was measured at its peak maximum to peak minimum (231.0–246.0 nm). D1 spectrum of AML was eliminated from the derivative ratio of laboratory-prepared mixture by subtraction of constant I then multiplication of the obtained spectra by divisor spectrum. The obtained spectra of D1 of TEL and CHT were divided by D1 spectra of 5 µg/mL TEL as divisor. The value of constant II (TEL/TEL$^{\prime})$ was measured in the plateau region that was parallel to the wavelength axis at 305.0–325.0 nm. The original D1 spectra of TEL were obtained by multiplication of the recorded constant II value with the divisor spectrum (D1 spectrum of TEL$^{\prime}).$ The TEL amplitude was measured at its peak minimum to peak maximum (282.5–313.0 nm). D1 spectrum of TEL was eliminated from the derivative ratio of TEL and CHT by subtraction of constant II value then multiplication of the obtained spectra by divisor spectrum (5.0 µg/mL TEL). The resulting spectrum was the corresponding original D1 spectra of CHT. The CHT amplitude was measured at its Peak maximum to zero baseline (287.0 nm). The steps that illustrate this method are presented in Fig. S2.

To optimize the SDS-CM method, various smoothing and scaling factors were evaluated. A smoothing factor Δ λ of 10 and a scaling factor of 10 were found to provide an appropriate signal-to-noise ratio, resulting in spectra with clear resolution and minimal noise interference. In the successive derivative subtraction method, ratio spectra were utilized, with the selected divisors carefully chosen to balance minimal noise and maximum sensitivity. Different divisors’ concentrations were tried including^5,10,20^ µg/mL for AML and TEL and it was found that 10.0 µg/mL AML and 5.0 µg/mL TEL gave the best results.

##### Validation of univariate spectrophotometric methods

Method validation was conducted according to ICH Q2(R1) guidelines^31^ to assess the performance characteristics of the proposed analytical methods (Table 1). The linearity and range were studied and characteristic parameters for regression equations were listed in Table 1. The proposed methods showed good linearity over concentration range of (5.0–40.0) µg/mL for TEL, (10.0–100.0) µg/mL for CHT and (5.0–25.0) µg/mL for AML.

**Table 1:** Validation parameters of the proposed univariate spectrophotometric methods for determination of TEL, CHT, and AML in pure forms.

Method	SRS			SDS		
Parameter	TEL	CHT	AML	TEL	CHT	AML
Range	5.0–40.0 μg/mL	10.0–100.0 μg/mL	5.0–25.0 μg/mL	5.0–40.0 μg/mL	10.0–100.0 μg/mL	5.0–25.0 μg/mL
Regression equations parameters						
Slope (b)^a^	0.0544	0.0055	0.0104	0.0033	0.0007	0.0024
Intercept (a)^a^	0.0232	0.0009	0.001	0.0006	0.00008	0.0002
Correlation coefficient (r)	0.9999	0.9999	0.9999	0.9999	0.9999	0.9999
Accuracy ^b^ (Mean ± SD)	100.35 ± 1.12	100.26 ± 1.02	99.73 ± 0.38	100.74 ± 0.50	100.23 ± 1.10	100.21 ± 0.47
Precision						
(%RSD)^c^	0.43	0.41	0.35	0.44	0.49	0.88
(%RSD)^d^	1.43	1.19	0.45	1.44	1.49	1.18
LOD^e^	0.228	0.579	0.326	0.404	0.574	0.376
LOQ^e^	0.689	1.753	0.987	1.225	1.738	1.139
Specificity^f^(Mean ± SD)	99.40 ± 0.89	99.22 ± 1.19	99.25 ± 1.20	100.01 ± 0.83	99.28 ± 1.07	100.00 ± 1.12

^a^Six calibration points in μg/mL, each conducted three times.

^b^Mean recovery ± SD for five concentration values in between the calibration points in μg/mL, each conducted three times.

^c^%RSD of three concentrations for TEL (10.0, 20.0, 30.0 μg/mL), CHT (10.0, 20.0, 30.0 μg/mL), and AML (5.0, 10.0, 15.0 μg/mL) repeated three times within the same day.

^d^%RSD of three concentrations for TEL (10.0, 20.0, 30.0 μg/mL), CHT (10.0, 20.0, 30.0 μg/mL), and AML (5.0, 10.0, 15.0 μg/mL) repeated three times in three successive days.

^e^ LOD and LOQ are calculated according to ICH, 3.3 × SD of the residuals/slope and 10 × SD of the residuals/slope, respectively.

^f^Mean Recovery ± SD for five concentration values for TEL, CHT, and AML in laboratory-prepared mixtures including dosage form ratio.

##### Accuracy

It was validated through recovery studies conducted at five concentration levels spanning the established linear range for each analyte. The obtained recoveries (Table 1) fell within acceptable limits, confirming the method’s high accuracy.

##### Precision

It was evaluated by measuring both intraday precision (repeatability) and intermediate precision. This was done by calculating the RSD% for three different concentrations through three replicate analyses of pure drugs conducted within the same day and over three consecutive days, respectively. The resulting values were found to be within the acceptable limit, all below 2%.

##### Specificity

The specificity of the proposed methods was assessed by testing them on laboratory-prepared mixtures with different ratios of TEL, CHT, and AML. As demonstrated in Table 1, the methods were found to be effective in accurately determining TEL, CHT, and AML. The LOD and quantitation LOQ were determined by using the slope of the response and the standard deviation. The LOD and LOQ values for the studied drugs are provided in Table 1.

### Multivariate chemometric methods

Various ratios of the three components were prepared following a five-level, three-factor calibration design, ensuring orthogonality of each component’s concentration relative to the others in the mixtures. Seventeen mixtures were used to build the regression models as a training set, while an additional eight samples were used as an external validation set, as presented in Table S1.

#### Full spectrum PLS model

Selecting the most informative variables or eliminating uninformative ones can further enhance the quality of the model. The goal of the PLS model was to design a calibration model that links the concentrations of the studied components to the latent variables of the data matrix. These latent variables are generated using concentration values, which are linear combinations of the original variables. PLS Model allows for the construction of a model specific to each analyte, potentially enhancing the model’s selectivity for that analyte. First, several preprocessing techniques—including mean-centering, autoscaling, and baseline correction—were applied to the raw spectral data. Among these, mean-centering produced the lowest RMSECV and RMSEP values, confirming it as the most robust and predictive approach. Other methods did not improve model performance, likely because the components were present at comparable scales within a similar matrix, reducing the need for variance scaling. Several cross-validation techniques were then tested, including random subsets, leave-one-out, contiguous blocks, and Venetian blinds. Among these, the leave-one-out method produced the most favorable outcomes, achieving the lowest RMSECV and the highest R-squared values^32,33^.

Optimizing the number of latent variables (LVs) is crucial for achieving accurate quantitation in the PLS model. An excessive number of LVs can lead to model overfitting, whereas an insufficient number may result in the loss of valuable data necessary for constructing the calibration model. Therefore, the optimum number of LVs contributing to the variance among the data was chosen according to Haaland and Thomas criteria^16^. The scanning range for the prepared samples spanned from 270.0 to 400.0 nm, with spectral data collected at intervals of 0.1 nm, resulting in a total of 1301 data points for each spectrum. Consequently, the spectral data matrix consists of 25 rows, corresponding to 25 samples, and 1301 columns, representing the wavelengths, yielding a matrix of dimensions 25 × 1301.

**Fig. 4:**
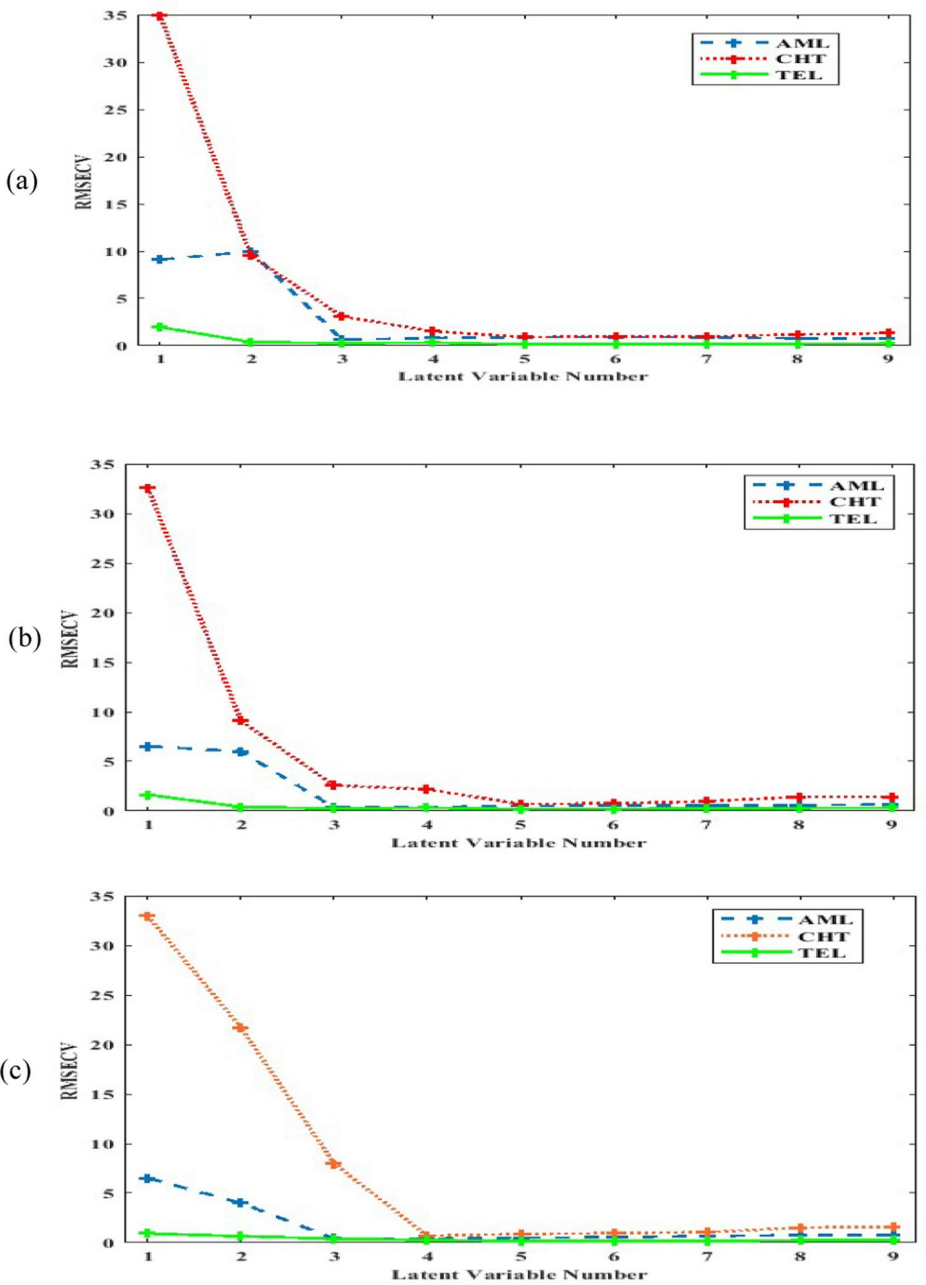
The optimum number of latent variables of TEL , CHT  and AML  for PLS model (**a**), iPLS model (**b**), GAPLS(**c**).

The root mean square error of cross-validation (RMSECV) was used as a diagnostic test to assess errors in the predicted concentrations. The optimal number of factors was determined as the one yielding the lowest RMSECV, with no significant differences observed beyond this point. TEL and CHT showed an optimal number of five latent variables (LVs), while AML showed an optimal number of four LVs, as illustrated in Fig. 4a.

Using the developed PLS model, the concentrations of the three components were determined, and the performance characteristics of the model were evaluated. The linear relationship was obtained by plotting the predicted concentration of the calibration set against their actual concentrations and the regression parameters (slope, intercept, correlation coefficient) were mentioned in Table 2. The developed PLS model was validated for predictive accuracy using an external validation set. The optimized PLS models were used to predict concentrations of the three analytes in the validation set and their performance characteristics were mentioned in Table 3.

**Table 2:** Statistical parameters for calibration sets using PLS, iPLS and GAPLS methods.

Parameters	PLS			iPLS			GAPLS		
	TEL	CHT	AML	TEL	CHT	AML	TEL	CHT	AML
Mean recovery %	100.16	100.65	101.37	100.05	100.06	100.06	100.04	100.12	100.08
SD	0.74	1.83	3.99	0.71	1.46	1.51	0.58	1.38	1.54
RSD	0.74	1.82	3.94	0.71	1.46	1.51	0.58	1.38	1.54
RMSEC	0.1013	0.6369	0.4682	0.1098	0.4057	0.2753	0.0878	0.4417	0.2736
RMSECV	0.1270	0.9253	0.9074	0.1739	0.7010	0.3607	0.1358	0.8680	0.4215
R^2 cal^	0.9999	0.9996	0.9956	1.000	0.9998	0.9988	1.000	0.9996	0.999

**Table 3:** Statistical parameters for validation sets using PLS, iPLS and GAPLS methods.

Parameters	PLS			iPLS			GAPLS		
	TEL	CHT	AML	TEL	CHT	AML	TEL	CHT	AML
Mean recovery %	100.04	101.71	108.56	99.40	100.75	98.22	99.77	100.37	98.18
SD	0.80	2.39	6.50	0.56	1.53	1.69	0.62	1.36	1.61
RSD	0.80	2.35	5.99	0.56	1.52	1.72	0.62	1.35	1.64
RMSEP	0.1955	0.6479	0.6164	0.2730	0.8516	0.1858	0.1957	0.6260	0.1770
Pred BIAS	− 0.0426	0.2460	0.5372	− 0.2004	0.2287	− 0.1252	− 0.0893	− 0.0368	− 0.1272
BCMSEP	0.0364	0.3593	0.0914	0.0344	0.6729	0.0188	0.0303	0.3905	0.0151
Q^2^	0.9997	0.9992	0.9690	0.9993	0.9990	0.9971	0.9997	0.9992	0.9974
R^2^	0.9997	0.9995	0.9942	0.9999	0.9992	0.9985	0.9998	0.9995	0.999

#### Wavelength selection

Variable selection is crucial in data analysis, as prior knowledge of which features are relevant or irrelevant is often unavailable in real-world scenarios. Thus, effective techniques are necessary to distinguish between relevant and irrelevant information and to eliminate redundant features that could impair the performance of the statistical model used to address the problem at hand. Typically, hundreds of wavelengths are considered, but only a few are truly informative, with many redundant variables included. Variable selection can significantly enhance the predictive ability of the chosen model, especially since the high correlation of consecutive variables in a spectrum often results in multi-collinearity^14^. Notable algorithms developed for variable selection include interval partial least squares (iPLS) and genetic algorithm-partial least squares (GA-PLS)^34^.

##### iPLS model

Recently, more advanced chemometric algorithms, such as iPLS, have been introduced for application across various numerical data sets. These algorithms offer the benefit of signal selection to enhance performance^35^. Interval PLS (iPLS) regression is a modified version of the PLS algorithm, where the sample spectrum is divided into smaller, equidistant intervals. iPLS is an interval selection method introduced by Nørgaard et al.^15^ as an alternative to using principal components or latent variables for dimensionality reduction in highly correlated spectral data. This method aims to enhance the predictive ability of the models and reduce time and costs by identifying critical regions, effectively managing the large amount of redundant information typically present in such data.

iPLS applies PLS to the selected intervals of variables with equal width, as well as to the full set of explanatory variables, using a consistent number of latent variables. An appropriate error metric, typically the root mean square error of cross-validation (RMSECV), is calculated for each model, and the region with the lowest error is selected. The primary advantage of this approach is its speed and the ease with which the results can be interpreted^15^. Forward iPLS starts from the interval granting the lowest error and then iteratively adds the interval whose addition implies the best predictive performances^36^. PLS regression is then applied to each interval, allowing for the optimization of prediction models by excluding subintervals that lack relevant information or contain interference. iPLS regression allows for the combination of these equidistant intervals to create regressions. Subsequently, the results are automatically presented as combinations of PLS component numbers and intervals. The RMSECV values for the best models are calculated, with these values primarily depending on the number and combination of intervals. The built iPLS model was applied to the studied mixture to identify the most informative regions, improving prediction accuracy, minimizing interference, and reducing the number of latent variables compared to PLS.

Several iPLS models were constructed using different numbers of subintervals and different interval sizes (window width in variables) along the full spectrum (270–400) nm. By comparing the different built models, it was found that model which composed of 40 subintervals and 10 interval size gave better RMSECV (0.514) and acceptable predictive recoveries of the three components as shown in Fig. S3. The final intervals for the model were selected based on their individual performance, retaining only those intervals where the local RMSECV was lower than that of the full-spectrum model. TEL and CHT showed optimum LVs of 5, while AML showed optimum LVs of 4 as shown in Fig. 4b. The raw number of variables was 1301 (270.0–400.0 nm), 0.1 nm interval. By using iPLS model, the number of variables decreased to 400 variables about (30% of original variables). The performance parameters of the developed model were listed on Tables 2 and 3.

##### GA-PLS model

The spectra of the calibration and validation sets were subjected to GA pre-processing. GA proved to be an effective technique for selecting the most significant wavelengths for the chemometric models. Uninformative variables were removed, and the selection of significant variables was successfully accomplished. Optimizing GA parameters is crucial for its effectiveness. The key parameters needing optimization include the number of wavelengths in a window, the maximum number of generations, the mutation rate, the percentage of genes involved at initiation, the breeding crossover rule, and the percentage of the population that remains constant at convergence. Additionally, user-selected parameters include the type of cross-validation (random or contiguous blocks), the maximum number of latent variables for PLS-1, the number of subsets for cross-validation, and the number of iterations for cross-validation at each generation. The GA parameters were adjusted as specified in Table S2.

GA procedure was applied to enhance the calibration quality. Uninformative variables were eliminated, and the variables selection was achieved^37^. The GA was run on the 1301 variables of TEL, CHT, and AML using PLS with the maximum number of latent variables that determined by cross-validation on the model containing the whole variables. The optimal latent variables were 5 for TEL and CHT and 4 for AML as presented in Fig. 4c. GA minimized the absorbance matrix to about 27% of the original matrix variables (352 variables for all components) as presented in Fig. S4. The statistical parameters for calibration and validation sets for GAPLS model were mentioned in Tables 2 and 3.

By comparing the different built models in iPLS, it was found that model which composed of 40 subintervals and 10-interval size achieved better RMSECV (0.514) and acceptable predictive recoveries. GA minimized the absorbance matrix to about 27% (352 variables) of the original matrix variables (1301). The built GAPLS model gave high performance and high predictive abilities with RMSECV (0.456).

##### Comparative study of the proposed chemometric methods

**Fig. 5:**
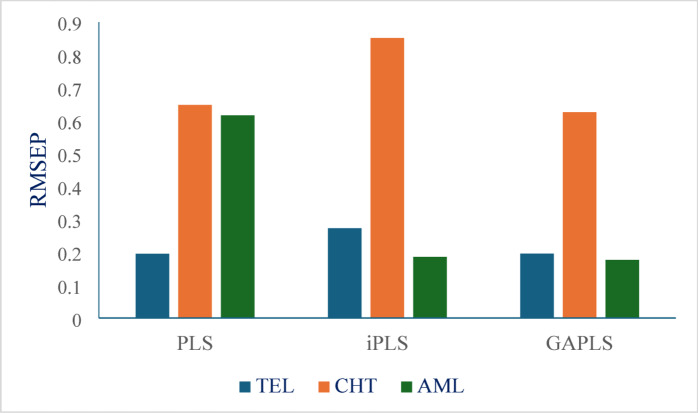
Comparison of RMSEP of the three components between the three proposed chemometric models.

- Calibration set performance
  Regarding the accuracy, the mean recovery percent of all built models are within the accepted range for all cited drugs. In PLS model, standard deviation of AML (3.99) is higher than in other two models. This is because PLS model failed in prediction of AML in some mixtures. High R^a^ values can coexist with poor prediction due to overfitting, as initially observed with the PLS model for AML. This was the primary rationale for employing variable selection techniques (iPLS& GAPLS). Comparing the three models, GAPLS model has the lowest SD/RSD for all cited drugs which indicate better precision. iPLS model outperforms PLS model for both CHT and TEL (SD of CHT = 1.46 in iPLS while 1.83 in PLS). GA-PLS has the lowest RMSEC for all cited drugs, suggesting better calibration fit. iPLS demonstrates improved generalizability for AML, with a lower RMSECV (0.3607 compared to 0.9074 in PLS), emphasizing the potential overfitting issue in the PLS model. All models achieve near-perfect R^2^ values (> 0.995), but GA-PLS and iPLS slightly outperform PLS for drugs CHT and AML (e.g., for Drug AML: R^2^ = 0.999 with GA-PLS vs. 0.9956 with PLS).
- Validation set performance
  Regarding accuracy, in case of AML, PLS model fails (Mean R% = 108.56%), while iPLS and GAPLS are in the accepted range (98.22–98.18%), demonstrating better robustness. In case of TEL and CHT, all models perform well, but GAPLS show least bias (CHT, Pred Bias = − 0.0368 vs. 0.2460 in PLS). GA-PLS maintain the lowest SD/RSD across all cited drugs (e.g. SD of TEL = 0.62 vs 0.80 in PLS). iPLS is slightly less precise for CHT (SD = 1.53 vs 1.36 in GAPLS). GAPLS has the lowest RMSEP for CHT and AML, confirming its predictive superiority. GAPLS attains the highest r^2^ for AML(0.999 vs 0.994 in PLS), reinforcing its stability. Figure 5 reveals the comparison of RMSEP of the three cited drugs between the three proposed chemometric methods. By comparing the three built models, it was found that PLS model serves as the most straightforward modeling approach but demonstrates limited robustness, particularly with complex analytes. For example, AML exhibits elevated RMSEP, standard deviation, and recovery bias under PLS modeling. Additionally, the considerable discrepancy between RMSEC and RMSEP suggests a tendency toward overfitting in such cases. iPLS enhances model performance by selecting informative spectral intervals, offering improvements over standard PLS. While generally outperformed by GA-PLS, iPLS remains advantageous when interpretability of specific spectral regions is essential, such as in the identification of functional groups.

GAPLS demonstrates the most favorable overall performance, achieving a well-balanced combination of accuracy, precision, and robustness. Although overfitting could theoretically arise if the algorithm selects an excessive number of variables, the current findings indicate stable validation metrics, reinforcing GA-PLS as a reliable and robust modeling strategy.

### Application to the pharmaceutical formulation and content uniformity

The developed univariate and multivariate models have been applied for the quantitative analysis of the cited medications in their combined formulation with no interference from excipients as presented in Table 4. Standard addition method was employed to validate the developed procedures. Content uniformity is the consistency of the amount of drug substances that are present in individual dosage units. Uniformity can be assessed using either content uniformity or weight variation methods. The content uniformity was applied according to USP where ten tablets were individually analyzed, yielding an average recovery between 98.50% and 101.5%. The acceptability constant (k) was set at 2.4, and the final acceptance value (AV) was calculated by multiplying the standard deviation of the result by this constant. As shown in Table 5, the proposed methods produced AV values below the USP limit of 15 (L1), confirming good content uniformity of the tested dosage units.

**Table 4:** Determination of TEL, CHT, and AML in pharmaceutical dosage form by the proposed spectrophotometric methods.

Pharmaceutical formulation				SRS method	SDS method	iPLS method	GAPLS method
Telma-ACT® Tablets (Each tablet was labelled to Contain 40 mg TEL, 12.5 mg CHT & 5 mg AML) Batch No. 30TTM008	Drug	Claimed (μg/mL)					
	TEL	40	%Found ± SD^a^	99.99 ± 1.13	99.15 ± 0.85	101.26 ± 0.62	100.14 ± 0.51
	Standard addition		%Recovery ± SD^b^	100.97 ± 0.90	100.01 ± 1.35	100.06 ± 0.92	100.49 ± 0.53
	CHT	12.5	%Found ± SD^a^	100.61 ± 1.61	100.34 ± 1.39	100.17 ± 0.78	100.56 ± 0.81
	Standard addition		%Recovery ± SD^b^	101.23 ± 0.51	99.91 ± 1.70	100.27 ± 0.77	100.36 ± 0.75
	AML	5	%Found ± SD^a^	100.37 ± 1.45	100.45 ± 1.15	101.22 ± 1.81	100.33 ± 1.33
	Standard addition		%Recovery ± SD^b^	100.24 ± 0.22	99.92 ± 1.39	100.46 ± 0.70	100.84 ± 0.67

^a^Average of five determinations.

^b^Average of three determinations.

**Table 5:** Results of content uniformity testing for determination of TEL, CHT, and AML in Telma-ACT® Tablets by the proposed spectrophotometric methods.

Telma-ACT® Tablet No.	Label claim (%)											
	TEL				CHT				AML			
	SRS	SDS	iPLS	GAPLS	SRS	SDS	iPLS	GAPLS	SRS	SDS	iPLS	GAPLS
1	101.32	98.55	100.54	100.47	100.84	101.94	99.46	99.46	100.15	99.14	99.49	99.04
2	100.27	98.79	101.65	99.55	102.10	99.43	100.04	100.04	99.04	101.31	101.05	100.25
3	98.75	100.12	101.59	100.40	98.89	99.66	101.00	101.00	101.92	100.90	103.11	101.71
4	99.81	100.53	99.92	99.88	99.20	103.09	99.74	101.13	100.19	102.13	100.85	101.50
5	99.80	98.56	98.87	99.10	103.42	98.51	100.39	102.03	97.87	100.49	97.91	101.73
6	101.42	99.24	99.96	99.97	99.05	99.66	98.53	100.66	98.21	102.87	98.37	98.76
7	100.82	99.58	99.49	97.76	98.33	100.80	100.36	97.96	101.92	98.85	98.58	99.51
8	100.17	100.76	98.43	100.00	101.96	99.54	100.29	99.08	99.42	101.39	97.82	99.78
9	99.94	100.61	99.96	98.65	100.51	101.94	102.50	100.37	100.58	98.61	97.90	97.55
10	100.96	98.79	100.94	99.11	100.84	99.66	101.29	100.77	100.15	100.90	97.80	97.83
Mean	100.33	99.15	100.14	99.49	100.52	100.34	100.36	100.25	99.95	100.66	99.29	99.77
SD	0.82	0.89	1.07	0.85	1.65	1.45	1.08	1.17	1.36	1.41	1.82	1.53
RSD%	0.82	0.90	1.07	0.85	1.64	1.45	1.08	1.17	1.36	1.40	1.83	1.53
AV*	1.97	2.14	2.57	2.04	3.96	1.08	2.59	2.81	3.26	3.38	4.37	3.67

*Acceptance value = 2.4 × SD with maximum allowed level (L1) is 15.

### Statistical comparison

A statistical comparison between the proposed spectrophotometric methods and the reported method^21^ demonstrated no significant difference, as evidenced by the data in Table S3. The calculated t-test and F-values were both lower than the corresponding tabulated values.

### Ecological assessment metrics

The findings of this study highlight a key insight into sustainability: a method cannot be considered truly sustainable based solely on its green attributes unless it also demonstrates strong performance and economic feasibility. In analytical chemistry, genuine sustainability is realized through a balanced integration of three essential dimensions—efficacy, environmental friendliness, and cost-effectiveness.

#### Analytical greenness metric (AGREE)

**Fig. 6:**
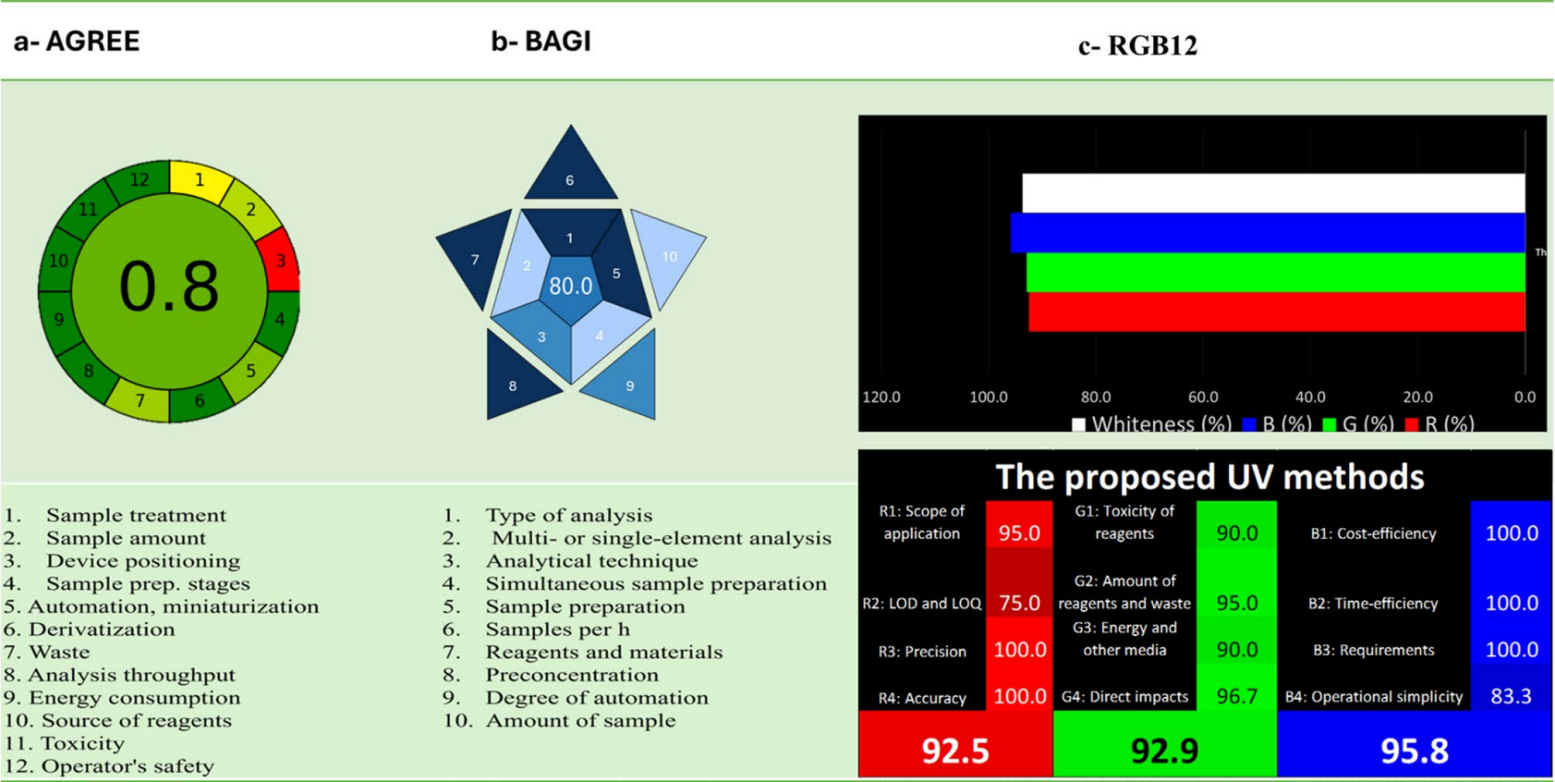
Ecological assessment including (**a**) AGREE (**b**) BAGI and (**c**) RGB12.

AGREE is a powerful tool used to evaluate the environmental friendliness of analytical methods both qualitatively and quantitatively. It provides a comprehensive assessment of the whole procedure. Its main advantages are ease of use, automation, and the generation of a numerical score, making it the most efficient method for adhering to the 12 principles of Green Analytical Chemistry^38–40^.When applied to the proposed spectrophotometric method, the AGREE tool produced a score of 0.80 (Fig. 6), reflecting strong compliance with green chemistry principles and highlighting the method’s environmental sustainability.

#### Blue applicability grade index (BAGI)

The Blue Applicability Grade Index (BAGI) is a recently developed metric aimed at evaluating the practical usability of analytical techniques in real-world applications. Designed to complement existing green metrics, BAGI focuses on the operational considerations emphasized in the framework of White Analytical Chemistry. The index incorporates ten evaluative criteria (e.g. ability to determine multiple analytes simultaneously, reagent and material requirements, and required sample volume). By evaluating these attributes, BAGI generates a visual representation in the form of an asteroid diagram, accompanied by a numerical score^38^. The color gradient of the BAGI pictogram visually conveys the extent to which an analytical method meets predefined criteria. Dark blue signifies a strong alignment, blue indicates moderate alignment, light blue reflects low alignment, and white denotes no alignment. The central numeric value represents the overall BAGI score, ranging from 25 to 100, summarizing the method’s practical applicability. The spectrophotometric method was evaluated using the BAGI tool, yielding a favorable score of 80, which indicates strong practical applicability, as illustrated in Fig. 6.

#### Whiteness assessment tool

White analytical chemistry (WAC) offers a comprehensive tool for assessing the sustainability of analytical methods by integrating three key criteria: method performance (red), environmental impact (green), and economic/practical considerations (blue). An Excel-based worksheet simplifies the evaluation, providing a final sustainability score out of 100, and a visual whiteness diagram illustrating the balance among these factors. In this study, the proposed methods demonstrated excellent sustainability with a red score of 92.5 (high accuracy and precision), green score of 92.9% (reduced solvent, energy and waste), and a blue score of 95.8% (cost and operational efficiency) as shown in Fig. 6. The overall whiteness score of 93.8% confirms the method’s strong sustainability profile.

#### Need, quality, and sustainability index (NQS)

In 2023, K. Kiwfo and colleagues enhanced the RGB12 Algorithm by developing the NQS indicator, a new framework designed to assess the sustainability of analytical methods in line with the United Nations Sustainable Development Goals (UN-SDGs)^29^. This multi-metric strategy is further strengthened by the introduction of NQS index, which combines societal relevance, analytical quality, and environmental impact into a single metric directly aligned with the United Nations Sustainable Development Goals^41–43^. Table 6 outlines the specific SDGs addressed by this research, underscoring their role in promoting environmentally responsible practices within the scientific community. The NQS model evaluates methods based on three key dimensions: Need, Quality, and Sustainability.

- *Need* emphasizes practical applicability and resource efficiency, drawing on Koel’s pyramid to classify methods according to their real-world demand and usability. This reflects the growing industry emphasis on both effectiveness and sustainability.
- *Quality* is assessed using the RGB12 Algorithm, with an overall score of 93.8% derived from the mean of the red (92.5%) (high accuracy and precision), green (92.9%) (reduced solvent, energy and waste), and blue (95.8%) (cost and operational efficiency) scores, as shown in Fig. 6, confirming the method’s strong sustainability profile.
- *Sustainability* evaluates a method’s alignment with the 17 UN-SDGs, providing a percentage score that quantifies its contribution to global sustainability efforts.

**Table 6:** Impact of eco-friendly spectrophotometric methods on UN-SDGs 3, 4, 5, 7, 9, 11, 12, 13, 14, 15, and 17.

SDG	Goal	Application(s)
	Good health and well-being	Developed innovative spectrophotometric methods for antihypertensive drugs’ analysis, supporting health monitoring and pharmaceutical quality control
	Quality education	Promotes scientific research and educational enrichment by advancing analytical chemistry knowledge and encouraging sustainable lab practices
	Gender equality	The research reflects inclusive collaboration among male and female scientists from diverse national backgrounds, promoting gender equality and inclusive practices in scientific research
	Affordable and clean energy	Spectrophotometric technique ensures minimal energy consumption, supporting sustainable and modern energy use in analytical instrumentation
	Industry, innovation, and infrastructure	The proposed spectrophotometric methods promote sustainable industrial innovation by reducing energy consumption, minimizing hazardous waste, and simplifying analytical procedures for broader implementation
	Sustainable cities and communities	The use of environmentally friendly and reusable spectrophotometric methods supports sustainable scientific practices, promotes efficient resource use, and reduces reliance on disposable materials in research environments, aligning with global community sustainability goals
	Responsible consumption and production	Although ethanol was used as the solvent, the proposed methods minimize solvent volumes and eliminate the need for toxic or chlorinated solvents, aligning with sustainable resource use and reduced environmental impact
	Climate action	The method reduces the use of hazardous chemicals and minimizes environmental pollution. Its low energy requirements and limited hazardous waste generation contribute to lowering the carbon footprint of analytical practices
	Life below water	The use of non-toxic reagent (ethanol) prevents chemical contamination of aquatic environments, thereby protecting marine ecosystems and supporting the health of marine life
	Life on land	Reducing hazardous chemicals in analytical methods helps protect terrestrial ecosystems from pollution and degradation
	Partnerships for the goals	Collaboration among researchers from different universities highlights national cooperation and joint efforts to advance sustainable practices in scientific research

By combining these three core pillars, the NQS index delivers a holistic, data-driven approach to sustainability assessment, with higher scores ensuring that eco-friendly claims are backed by transparent and balanced criteria.

The application of the NQS index to this study yielded highly significant results. The evaluated method achieved:

- A perfect 100% score in the “Need” dimension, confirming its essential role and strong market demand
- An exceptional 93.8% rating for “Quality”, demonstrating both superior analytical performance and negligible environmental impact (RGB12 tool evaluation results in Fig. 6)
- A 65% “Sustainability” score, indicating strong but incomplete alignment with the SDGs as shown in Table 6.

This reflects its substantial contribution to sustainable practices while acknowledging inherent limitations—such as indirect relevance to certain goals (e.g., poverty reduction) within pharmaceutical contexts. The overall NQS score was 86%, derived from the average of the three evaluated aspects: need (100%), quality (93.8%), and sustainability (65%). This score underlines the method’s high sustainability performance, balanced by the recognition that not all SDGs are equally applicable across analytical domains.

## Conclusion

The developed univariate and multivariate spectrophotometric methods gave simple, cost-effective and accurate simultaneous determination of TEL, CHT and AML in their combined dosage form. The developed univariate methods included the Successive Ratio Subtraction coupled with Constant Multiplication and the Successive Derivative Subtraction coupled with Constant Multiplication. These methods allowed selective quantification under optimized conditions. Multivariate analysis was achieved using two models: Interval-PLS and Genetic Algorithm-PLS. These methods effectively resolved spectral overlaps, improving analytical efficiency. It was found that GA-PLS demonstrates the most favorable overall performance, achieving a well-balanced combination of accuracy, precision, and robustness. The univariate method offers an advantage over the multivariate approach, as it enables quick quantitative analysis of solutions without requiring multiple sample preparations or specialized software.

A statistical comparison with the reported method revealed no significant differences, thereby confirming the accuracy and reliability of the proposed methods. Validation according to ICH guidelines demonstrated good precision, accuracy, and linearity for both approaches. Additionally, ecological assessments using AGREE, BAGI, WAC and NQS highlighted their environmental benefits compared to other analytical techniques. This research exemplifies the integration of environmental sustainability with scientific innovation in analytical chemistry, emphasizing the value of aligning with the United Nations Sustainable Development Goals (UN-SDGs). The proposed methods were effectively utilized for the quantitative assay of the formulation as well as for verifying the content uniformity in accordance with the regulatory standards. The proposed methods offer sustainable and statistically-validated tools for routine pharmaceutical quality control.

## Supplementary Information

Supplementary Information.

## Data Availability

All data generated or analysed during this study are included in this published article.

## References

[1] Kitt, J., Fox, R., Tucker, K. L. & McManus, R. J. New approaches in hypertension management: A review of current and developing technologies and their potential impact on hypertension care. *Curr. Hypertens. Rep.* **21**, 1–8 (2019). 10.1007/s11906-019-0949-4 PMC6483962 31025117

[2] Atkins, E. R. & Chow, C. K. Low-dose combination therapy for initial treatment of hypertension. *Curr. Hypertens. Rep.* **22**, 1–5 (2020). 32852644 10.1007/s11906-020-01069-7

[3] Whelton, P. K., Carey, R. M., Aronow, W. S., Acc/aha/aapa/abc/acpm/ags/APhA/ASH/ASPC/nma/pcna guideline for the prevention, Detection, evaluation, and management of high blood pressure in adults: A Report of the American College of Cardiology/American heart Association. Task force on clinical practice guidelines. *J. Am. Coll. Cardiol.-2017.-Nov 13. Пoчки*. **7**(1):68–74 (2018). 10.1016/j.jacc.2017.11.006 29146535

[4] Williams, B. et al. 2018 ESC/ESH guidelines for the management of arterial hypertension: The task force for the management of arterial hypertension of the European Society of Cardiology (ESC) and the European Society of Hypertension (ESH). *Eur. Heart J.* **39**(33), 3021–3104 (2018). 30165516 10.1093/eurheartj/ehy339

[5] Sagarad, S. V., Kerure, S. B., Kumar, C. & Ramakrishna, M. The antihypertensive efficacy of chlorthalidone and telmisartan in Indian hypertensive patients who were uncontrolled with hydrochlorothiazide and telmisartan combination-A prospective and an open label study. *J. Clin. Diag. Res. JCDR.* **7**(4), 687 (2013). 10.7860/JCDR/2013/5437.2882 PMC3644445 23730647

[6] British Pharmacopoeia, Seventh ed., *Stationery office*, London (2013).

[7] USP42-NF37, United States Pharmacopoeia. In *R. United States Pharmacopoeial Convention Inc.*, USA. (Ed.) 2019.

[8] Panda, M., Dadi, V., Yarraguntla, S. R. & Rao, V. P. K. RP-HPLC method for determination of azelnidipine and telmisartan in pharmaceutical dosage form. *Res. J. Pharm. Technol.* **16**(2), 509–513 (2023).

[9] Yenduri, S., Sulthana, H. & Koppuravuri, N. P. Sustainablity evaluation of existed HPLC based analytical methods for quantification of amlodipine besylate and telmisartan using RGB model: A whiteness approach. *Green Anal. Chem.* **6**, 100074 (2023).

[10] Raveendran, K. Simultaneous estimation of cilnidipine hydrochloride and chlorthalidone in its combined dosage form by absorbance ratio method. *World* **2**(3), 28–39 (2023).

[11] Jeelani, S. & Kouznetsova, N. A new stability-indicating HPLC-UV method for determination of amlodipine besylate and its impurities in drug substance. *Heliyon* **9**(9), e19993 (2023). 37809728 10.1016/j.heliyon.2023.e19993 PMC10559668

[12] Lotfy, H. M., Tawakkol, S. M., Fahmy, N. M. & Shehata, M. A. Successive spectrophotometric resolution as a novel technique for the analysis of ternary mixtures of pharmaceuticals. *Spectrochim. Acta Part A Mol. Biomol. Spectrosc.* **121**, 313–323 (2014). 10.1016/j.saa.2013.10.090 24263128

[13] Elghobashy, M. R., Bebawy, L. I., Shokry, R. F. & Abbas, S. S. Successive ratio subtraction coupled with constant multiplication spectrophotometric method for determination of hydroquinone in complex mixture with its degradation products, tretinoin and methyl paraben. *Spectrochim. Acta Part A Mol. Biomol. Spectrosc.* **157**, 116–123 (2016). 10.1016/j.saa.2015.12.019 26745510

[14] Arboretti, R., Ceccato, R., Pegoraro, L. & Salmaso, L. Interval selection: A case-study-based approach. *Appl. Stoch. Model. Bus. Ind.* **37**(5), 926–941 (2021).

[15] Nørgaard, L. et al. Interval partial least-squares regression (i PLS): A comparative chemometric study with an example from near-infrared spectroscopy. *Appl. Spectrosc.* **54**(3), 413–419 (2000).

[16] Haaland, D. M. & Thomas, E. V. Partial least-squares methods for spectral analyses. 1. Relation to other quantitative calibration methods and the extraction of qualitative information. *Anal Chem.* **60**(11), 1193–1202 (1988).

[17] Reed, P., Minsker, B. & Goldberg, D. E. Designing a competent simple genetic algorithm for search and optimization. *Water Resour. Res.* **36**(12), 3757–3761 (2000).

[18] Kamel, M. A., Nessim, C. K., Michael, A. M., Abbas, S. S. & Marzouk, H. M. A sustainable HPLC method coupled with diode array detection for versatile quantification of telmisartan, chlorthalidone and amlodipine in a fixed-dose antihypertensive formulation and dissolution studies. *BMC Chem.* **18**(1), 166 (2024). 39267180 10.1186/s13065-024-01276-2 PMC11391801

[19] Palakurthi, A. K., Dongala, T., Yalavarthi, R. K. & Anireddy, J. QbD-based development of an extraction procedure for simultaneous quantification of telmisartan, amlodipine besylate and chlorthalidone in combination complex matrix formulation. *Biomed. Chromatogr.* **34**(2), e4755 (2020). 31755118 10.1002/bmc.4755

[20] Chaudhary, B. R. & Dave, J. B. Estimation of telmisartan, amlodipine and chlorthalidone in bulk and fixed dose combination using stability indicating high performance thin layer chromatography. *Indo Global J. Pharm. Sci.* **10**(3), 6–20 (2020).

[21] Sanap, R. M., Wavhale, S. R., Kunjir, V. V. & Shete, R. V. Analytical method development and validation for telmisartan, chlorthalidone and amlodipine by uv-spectroscopic method. *Res. J. Pharm. Technol.* **14**(11), 6049–6054 (2021).

[22] Attala, K. & Elsonbaty, A. Advanced eco-friendly UV spectrophotometric approach for resolving overlapped spectral signals of antihypertensive agents in their binary and tertiary pharmaceutical dosage form. *Spectrochimica Acta A.* **258**, 119855 (2021). 10.1016/j.saa.2021.119855 33964634

[23] Prajapati, P., Patel, A. & Shah, S. Simultaneous estimation of telmisartan, chlorthalidone, amlodipine besylate and atorvastatin by RP-HPLC method for synchronous assay of multiple FDC products using analytical FMCEA-based AQbD approach. *J. Chromatogr. Sci.* **61**(2), 160–171 (2023). 35446938 10.1093/chromsci/bmac030

[24] Mhaske, R., Garole, D., Mhaske, A. & Sahasrabudhe, S. RP-HPLC method for simultataneous determination of amlodipine besylate, valsartan, telmisartan, hydrochlorothiazide and chlorthalidone: Application to commercially available drug products. *Int. J. Pharm. Sci. Res.* **3**(1), 141 (2012).

[25] Abbas, A. E. F. et al. Simultaneously quantifying a novel five-component anti-migraine formulation containing ergotamine, propyphenazone, caffeine, camylofin, and mecloxamine using UV spectrophotometry and chemometric models. *BMC Chem.* **18**(1), 233 (2024). 39568060 10.1186/s13065-024-01339-4 PMC11580349

[26] Pena-Pereira, F., Wojnowski, W. & Tobiszewski, M. AGREE—Analytical GREEnness metric approach and software. *Anal. Chem.* **92**(14), 10076–10082 (2020). 32538619 10.1021/acs.analchem.0c01887 PMC7588019

[27] Manousi, N., Wojnowski, W., Płotka-Wasylka, J. & Samanidou, V. Blue applicability grade index (BAGI) and software: A new tool for the evaluation of method practicality. *Green Chem.* **25**(19), 7598–7604 (2023).

[28] Nowak, P. M., Wietecha-Posłuszny, R. & Pawliszyn, J. White analytical chemistry: An approach to reconcile the principles of green analytical chemistry and functionality. *TrAC, Trends Anal. Chem.* **138**, 116223 (2021).

[29] Kiwfo, K. et al. A new need, quality, and sustainability (NQS) index for evaluating chemical analysis procedures using natural reagents. *Microchem. J.* **193**, 109026 (2023).

[30] Carlsen, L. & Bruggemann, R. The 17 United Nations’ sustainable development goals: A status by 2020. *Int. J. Sust. Dev. World* **29**(3), 219–229 (2022).

[31] International Council for Harmonisation of Technical Requirements for Pharmaceuticals for Human Use (ICH), Validation of analytical procedures: Text and methodology. *Q2 (R1)*. **1**(20):05 (2005).

[32] Parisotto, G. et al. Total acid number determination in residues of crude oil distillation using ATR-FTIR and variable selection by chemometric methods. *Energy Fuels* **24**(10), 5474–5478 (2010).

[33] Al Kamaly, O. et al. D-optimal candexch algorithm-enhanced machine learning UV-spectrophotometry for five-analyte determination in novel anti-glaucoma formulations and ocular fluids: Four-color sustainability framework with NQS assessment and UN-SDG integration. *BMC Chem.* **19**(1), 198 (2025). 40616089 10.1186/s13065-025-01572-5 PMC12232207

[34] Abbas, A. E. F. et al. Application of machine learning assisted multi-variate UV spectrophotometric models augmented by kennard stone clustering algorithm for quantifying recently approved nasal spray combination of mometasone and olopatadine along with two genotoxic impurities: Comprehensive sustainability assessment. *BMC Chem.* **19**(1), 98 (2025). 40234857 10.1186/s13065-025-01391-8 PMC12001563

[35] Hegazy, M. A., Boltia, S. A., Fayed, A. S. & Musaed, A. Advanced chemometrics manipulation of UV-spectroscopic data for determination of three co-formulated drugs along with their impurities in different formulations using variable selection and regression model updating. *Spectrochim. Acta Part A Mol. Biomol. Spectrosc.* **202**, 359–367 (2018). 10.1016/j.saa.2018.05.038 29803974

[36] Zou, X., Zhao, J. & Li, Y. Selection of the efficient wavelength regions in FT-NIR spectroscopy for determination of SSC of ‘Fuji’apple based on BiPLS and FiPLS models. *Vib. Spectrosc.* **44**(2), 220–227 (2007).

[37] Leardi, R. Application of genetic algorithm–PLS for feature selection in spectral data sets. *J. Chemom.* **14**(5–6), 643–655 (2000).

[38] Lotfy, H. M., Obaydo, R. H. & Nessim, C. K. Spider chart and whiteness assessment of synergistic spectrophotometric strategy for quantification of triple combination recommended in seasonal influenza–Detection of spurious drug. *Sustainable Chemistry and Pharmacy.* **32**, 100980 (2023).

[39] Marzouk, H. M., Ayish, N. S., El-Zeany, B. A. & Fayed, A. S. Eco-friendly chromatographic platforms for simultaneous determination and impurity profiling of an antihypertensive ternary pharmaceutical mixture. *Sustain. Chem. Pharm.* **32**, 100978 (2023).

[40] Michael, A. M., Lotfy, H. M., Rezk, M. R. & Nessim, C. K. Development and evaluation of chemometric models for the estimation of sumatriptan in the presence of naproxen and a degradation product using UV spectrophotometry. *J. AOAC Int.* **107**(5), 749–760 (2024). 38730542 10.1093/jaoacint/qsae041

[41] Chanduluru, H. K., Sugumaran, A., Parvathi Kannaiah, K., Obaydo, R. H. & Mahmoud, L. H. Aligning drug analysis with SDGs: Spectrophotometric methods for triple antihypertensive drug using propylene carbonate and statistical Dixon’s and Grubb’s tests. *Green Chem. Lett. Rev.* **18**(1), 2510297 (2025).

[42] Saleh, A. M., Hassan, R. Y. A., Badawey, A. M. & Marzouk, H. M. Software-assisted evaluation of sustainable chemistry innovations: A critical analytical review of viability assays incorporating diseases’ biomarkers with greenness, blueness, and whiteness computational metrics. *Microchem. J.* **215**, 114437 (2025).

[43] Abdel-Hameed, R. et al. Electrochemical platform with Ag/ZnO nanorods for green, blue, and white determination of the newly approved drug roxadustat in pharmaceuticals and plasma: NQS assessment and UN-SDGs alignment. *Microchem. J.* **212**, 113326 (2025).

