# Supplementary material for: Univariate and multivariate signal processing spectrophotometric determination of an antihypertensive combination in line with the United Nations sustainable development goals
Source: Sci Rep. 2025 Oct 31;15:38103. doi: 10.1038/s41598-025-22700-0 (PMC12578892; doi:10.1038/s41598-025-22700-0)
Supplement: Supplementary file 1 — Supplementary Information. [file 41598_2025_22700_MOESM1_ESM.docx]

Supplementary material

For

**Univariate and multivariate signal processing spectrophotometric determination of an antihypertensive combination in line with the United Nations sustainable development goals**

***Mona A. Kamel ^a,b^, Hoda M. Marzouk ^c*^, Adel M. Michael ^b,d^, Samah S. Abbas ^c^, Christine K. Nessim ^b^***

*^a^ Postgraduate program, in Pharmaceutical Analytical Chemistry, Faculty of Pharmacy, Cairo University, Kasr El-Aini Street, Cairo 11562, Egyp^t^*

*^b^ Chemistry Department, Faculty of Pharmacy, Ahram Canadian University, 6th of October City, 12566, Cairo, Egyp^t.^*

*^c^ Pharmaceutical Analytical Chemistry Department, Faculty of Pharmacy, Cairo University, Kasr El-Aini Street, Cairo 11562, Egypt*

*^d^ Casual Academic, Charles Sturt University, Australia*


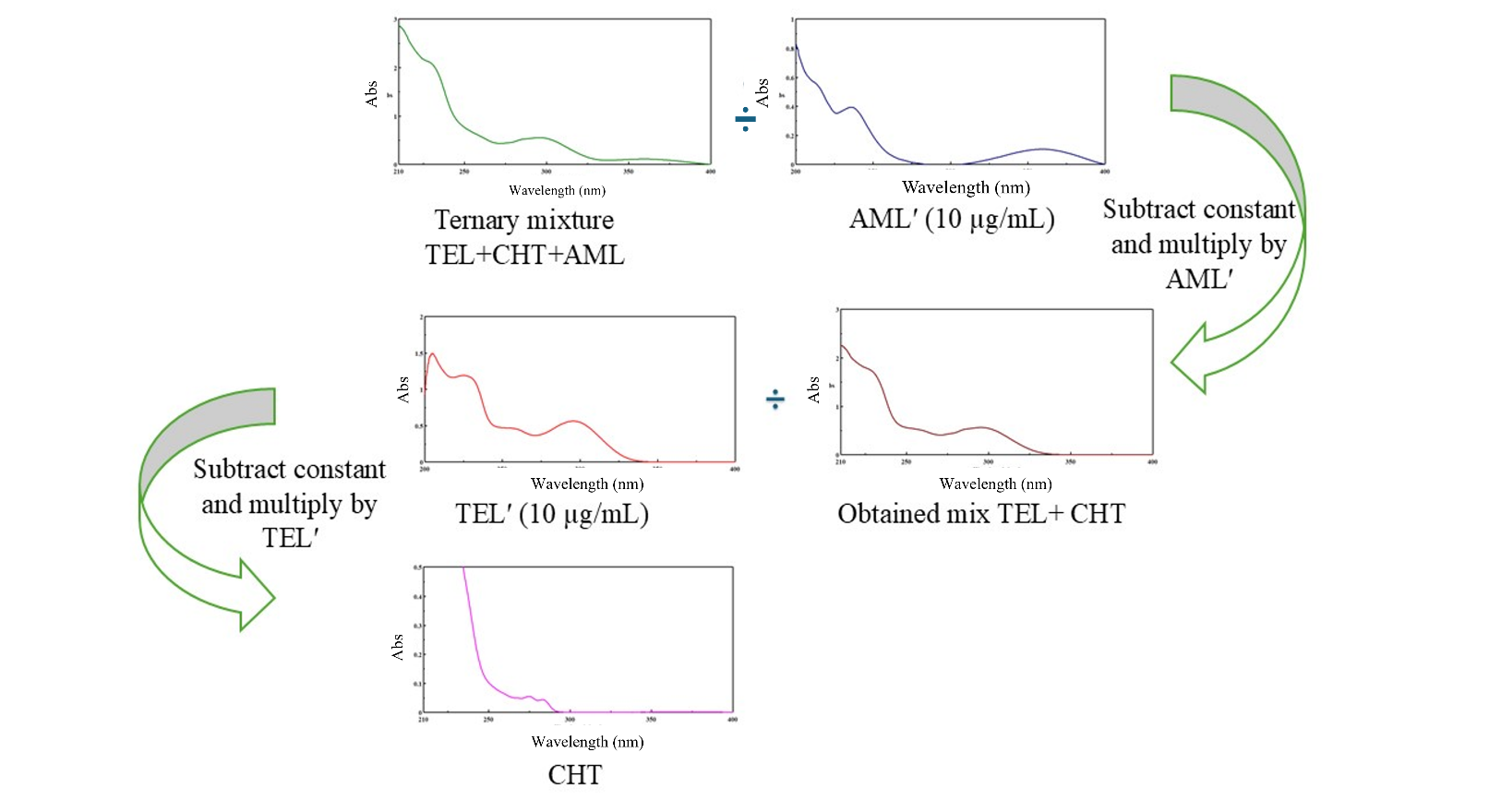


**Fig. S1.** Steps of Successive ratio subtraction method coupled with constant multiplication method.


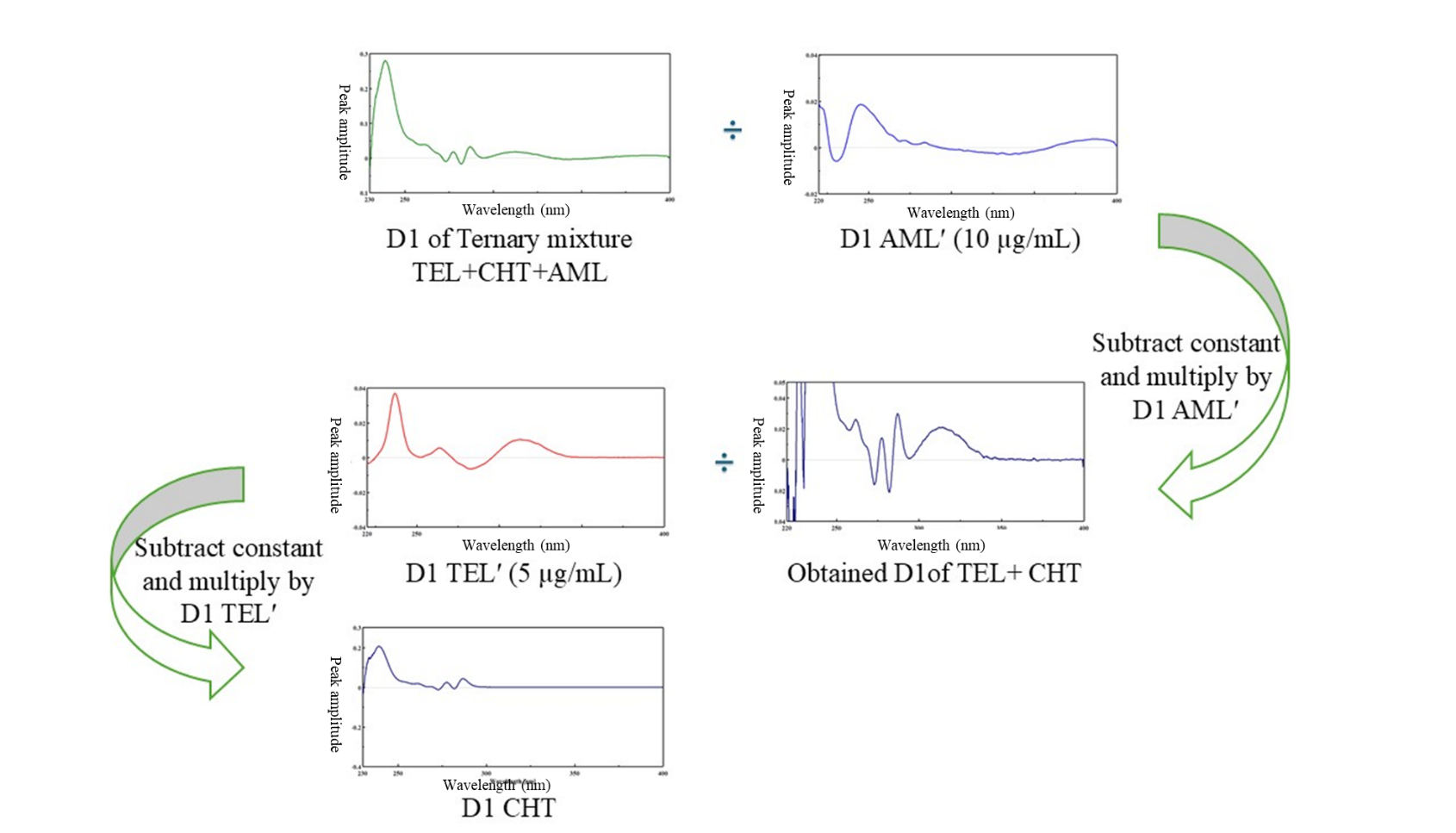


**Fig. S2.** Steps of Successive derivative subtraction coupled with constant multiplication method.


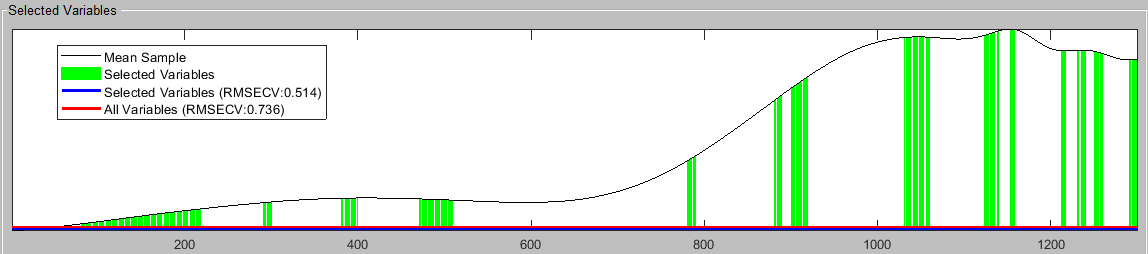


**Fig. S3.** Selected variables for proposed iPLS model.


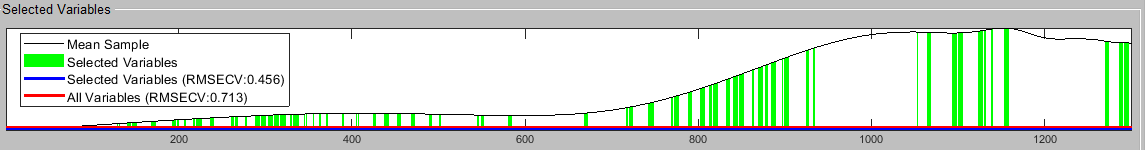


**Fig. S4**. Selected variables for the proposed GAPLS model.

**Table S1.** Concentrations of TEL, CHT, and AML in calibration and validation sets.

| **Mix No.** | **Levels** | | | **Concentration (μg/mL)** | | |
| --- | --- | --- | --- | --- | --- | --- |
|  | **AML** | **CHT** | **TEL** | **AML** | **CHT** | **TEL** |
| **1 ^b^** | 0 | 0 | 0 | 15 | 50 | 24 |
| **2 ^a^** | 0 | -2 | -2 | 15 | 10 | 8 |
| **3 ^b^** | -2 | -2 | 2 | 5 | 10 | 40 |
| **4 ^a^** | -2 | 2 | -1 | 5 | 90 | 16 |
| **5^a^** | 2 | -1 | 2 | 25 | 30 | 40 |
| **6^a^** | -1 | 2 | 0 | 10 | 90 | 24 |
| **7^a^** | 2 | 0 | -1 | 25 | 50 | 16 |
| **8^a^** | 0 | -1 | -1 | 15 | 30 | 16 |
| **9^b^** | -1 | -1 | 1 | 10 | 30 | 32 |
| **10^b^** | -1 | 1 | 2 | 10 | 70 | 40 |
| **11^a^** | 1 | 2 | 1 | 20 | 90 | 32 |
| **12^a^** | 2 | 1 | 0 | 25 | 70 | 24 |
| **13^a^** | 1 | 0 | 2 | 20 | 50 | 40 |
| **14^a^** | 0 | 2 | 2 | 15 | 90 | 40 |
| **15^a^** | 2 | 2 | -2 | 25 | 90 | 8 |
| **16^a^** | 2 | -2 | 1 | 25 | 10 | 32 |
| **17^b^** | -2 | 1 | -2 | 5 | 70 | 8 |
| **18^a^** | 1 | -2 | 0 | 20 | 10 | 24 |
| **19 ^b^** | -2 | 0 | 1 | 5 | 50 | 32 |
| **20^a^** | 0 | 1 | 1 | 15 | 70 | 32 |
| **21^a^** | 1 | 1 | -1 | 20 | 70 | 16 |
| **22^a^** | 1 | -1 | -2 | 20 | 30 | 8 |
| **23^b^** | -1 | -2 | -1 | 10 | 10 | 16 |
| **24^b^** | -2 | -1 | 0 | 5 | 30 | 24 |
| **25^a^** | -1 | 0 | -2 | 10 | 50 | 8 |

^a^ Calibration set ^b^ Validation set

**Table S2.** Genetic Algorithm Parameters Utilized in the Proposed Analytical Approach

| **Parameter** | **Value** |
| --- | --- |
| Population size | 100 |
| Maximum generations | 100 |
| Mutation rate | 0.005 |
| The number of variables in a window (window width) | 4 |
| Percent of population the same at  Convergence | 50 |
| % wavelengths used at initiation | 30 |
| Crossover type | double |
| Maximum number of latent variables | AML (4)  TEL (5)  CHT (5) |
| Cross validation | Random |
| Number of subsets to divide  data into for cross validation | 4 |
| Number of iterations for cross validation at each generation | 2 |

**Table S3.** Statistical analysis of the results obtained by the proposed methods and the reported method for the determination of Telmisartan, Chlorthalidone and Amlodipine in their pure forms [21]**.**

| Method  Parameters | TEL | | | | | CHT | | | | | | AML | | | | | |
| --- | --- | --- | --- | --- | --- | --- | --- | --- | --- | --- | --- | --- | --- | --- | --- | --- | --- |
|  | **SRS** | **SDS** | **iPLS** | **GAPLS** | **Reported** | | **SRS** | **SDS** | **iPLS** | **GAPLS** | **Reported** | | **SRS** | **SDS** | **iPLS** | **GAPLS** | **Reported** |
| Mean | 99.90 | 100.49 | 100.05 | 100.04 | 99.99 | | 100.01 | 100.51 | 100.06 | 100.12 | 99.99 | | 99.59 | 100.21 | 100.06 | 100.08 | 100.15 |
| SD | 0.70 | 0.54 | 0.71 | 0.58 | 1.19 | | 1.00 | 1.17 | 1.46 | 1.38 | 0.71 | | 0.60 | 0.91 | 1.51 | 1.54 | 1.32 |
| n | 6 | 6 | 17 | 17 | 6 | | 6 | 6 | 17 | 17 | 6 | | 6 | 6 | 17 | 17 | 6 |
| Variance | 0.49 | 0.29 | 0.50 | 0.34 | 1.43 | | 1.00 | 1.36 | 2.14 | 1.91 | 0.50 | | 0. 36 | 0.83 | 2.28 | 2.37 | 1.74 |
| Student’s t-test * | 0.187  (2.228) | 1.229  (2.228) | 0.110  (2.447) | 0.099  (2.447) | …… | | 0.285  (2.228) | 1.016  (2.228) | 0.138 (2.101) | 0.283  (2.101) | ……. | | 0.893  (2.228) | 0.096  (2.228) | 0.138  (2.228) | 0.111  (2.228) | ……. |
| F value * | 2.918  (5.05) | 4.931  (5.05) | 2.860  (4.604 | 4.206  (4.604) | ……. | | 2.000  (5.05) | 2.720  (5.05) | 4.28  (4.604) | 3.82  (4.604) | ……. | | 4.833  (5.05) | 2.096  (5.05) | 1.310  (4.604) | 1.362  (4.604) | ……. |

^*^ The values in the parenthesis are the corresponding theoretical values of t and F at P = 0.05.
